# Description and Validation of Flow-Through Chambers of Respirometry for Measuring Gas Exchange in Animal Trials

**DOI:** 10.3390/ani13162675

**Published:** 2023-08-19

**Authors:** Rony Riveros Lizana, Rosiane de Souza Camargos, Raully Lucas Silva, Bruno Balbino Leme, Nilva Kazue Sakomura

**Affiliations:** Faculty of Agricultural and Veterinary Sciences, Sao Paulo State University, Via de Acesso Prof. Paulo Donato Castellane s/n, Jaboticabal 14884-900, SP, Brazil; ronriveros@gmail.com (R.R.L.); rosiane.ifmg@gmail.com (R.d.S.C.);

**Keywords:** farm animals, gas exchange, energy expenditure, metabolic rate, respirometric chambers

## Abstract

**Simple Summary:**

The indirect calorimetry method has been widely used for a long time in the study of energy metabolism in animals, and it remains an important tool for investigating energy metabolism and feed values. However, to ensure the quality of research data, it is necessary to standardize the calibration procedure. This paper presents a detailed procedure for calibrating and calculating indirect calorimetry data.

**Abstract:**

Indirect calorimetry (IC) is a widely used method to study animal energy metabolism by measuring gas exchange. The accuracy of IC depends on detecting variations in signals reflecting the metabolic response, which can be challenging due to measurement noise and external factors. This study proposes a methodology to validate IC systems, including an easy-to-use spreadsheet for data computing, to verify accuracy and detect whole-system leaks. We conducted a recovery test using a simulation of CO_2_ dynamics in MS Excel and injecting a known CO_2_ concentration into four respirometry chambers. The thought flow rate of CO_2_ was observed and compared to the expected rate from the simulation. Data filtering and computing, including a detailed calculation of signals calibration, Bartholomew transformation, and noise reduction, was developed to obtain the gas exchange and heat production parameters using an open-circuit IC system. The results from the recovery test in our system show that the proposed methodology is accurate and precise. The proposed methodology and recovery test can be used to standardize the validation of IC systems together with adequate data computing, providing accurate measurements of animal energy metabolism in different environmental conditions and energy utilization from feeds.

## 1. Introduction

Indirect calorimetry (IC) is a widely utilized method for investigating the energy metabolism of animals and humans. It involves the direct measurement of gas exchange (O_2_ and CO_2_) to calculate heat production (HP) based on the volumetric stoichiometry principle of oxygen consumed (VO_2_), carbon dioxide produced (VCO_2_), and heat released during the oxidative process [1]. Recently, IC has garnered interest among animal researchers as it supports studies on energy metabolism and the development of net energy systems [2,3,4,5].

IC enables accurate assessment of animal HP under different environmental conditions, energy utilization from feeds, and other temporal measurements [2,3,6]. Advancements in gas analyzer technologies, data acquisition systems, and computing power have enhanced measurement accuracy over the years [7,8]. However, ensuring the quality and accuracy of results necessitates the involvement of trained technicians to conduct biological trials, handle equipment appropriately, perform calibration procedures, and acquire data adequately [9].

The accuracy of an IC system depends on its ability to detect and record variations in signals reflecting the metabolic or physiological response of the animal, which the researcher interprets later [10,11]. Various procedures have been developed to assess IC accuracy, including alcohol or propane gas combustion, alcohol evaporation, continuous gas injection, and specific volume gas injection. However, some methods have limitations, such as low sensitivity, complex calculations, expensive materials or substances, and time-consuming procedures. Consequently, the recovery test should be flexible to accommodate the diversity of IC systems and research objectives while simulating the experimental conditions of a biological trial, considering the flow used and the observed delta of CO_2_ and O_2_ during animal chamber measurements.

Inaccurate measurements or undetected system leaks within the IC setup can lead to misleading results, compromising the validity of animal energy metabolism studies. Erroneous measurements may result in the overestimation or underestimation of energy utilization from feeds, potentially leading to suboptimal estimations of energy requirements. Therefore, the calibration procedure should align with the actual outgoing gas concentration the animal releases.

The open-circuit system integrated into the trough airflow of pull-mode calorimetric chambers is commonly employed in farm animal trials [2,3,6,9,12]. This system measures the concentration of gases (O_2_ and CO_2_) and their rate of change, considering the airflow from the atmosphere into the chamber. Subsequently, the volume of gas exchanged (VO_2_ and VCO_2_) over time can be calculated. However, before obtaining the final HP value, a series of computations are applied to preserve signals associated with the metabolic response, identify atypical signals resulting from extraneous factors, and suppress measurement noise [12,13,14].

This paper aims to establish a standardized methodology for validating IC systems, ensuring their accuracy, and detecting whole-system leaks. We propose using an easy-to-use spreadsheet for data acquisition and final HP calculation based on Brower’s [1] fundamental equation. This approach yields an improved transient response and effectively suppresses measurement noise.

## 2. Materials and Methods

### 2.1. General Description of the Flow-Through IC System

**Figure 1:**
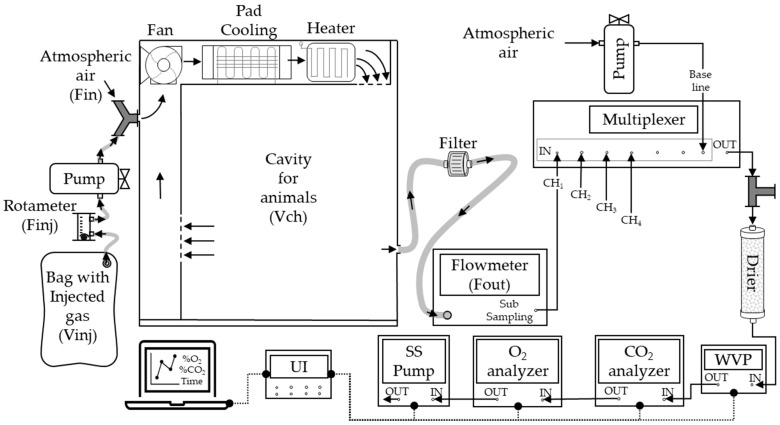
Scheme of multiple flow-through respirometry systems and coupling to the gas for injection test. Fin: ingoing flow. Fout: outgoing flow. Finj: injection flow. CH_i_: chambers (i = 1, 2, 3, 4). WVP: water vapor pressure analyzer. The arrows represent the airflow direction (→). Data transference line (●---●).

We utilized an open-circuit indirect calorimetry (IC) system capable of connecting six chambers, as illustrated in Figure 1. Each chamber had an identical geometric volume (Vch) of 0.980 m^3^ (dimensions: 100 cm × 100 cm × 98 cm). Inside each chamber, a temperature control system comprising a heater and a cooler was implemented to maintain air temperature of 24 ± 1.0 °C and a relative humidity of 60% throughout the experimental trials.

The experimental setup consisted of air-conduction components, analyzers, and data acquisition equipment. Mass flow pumps (FK-100, Sable System, Las Vegas, NV, USA) were connected to each chamber and operated at a flow rate of 20 L/min, matching the measurements conducted on the animals. To ensure a consistent sample flow through the gas analyzers, an air sample of 160 ± 2.0 mL/min was extracted from each flow pump using a sub-sampler pump (SS4, Sable System, Las Vegas, NV, USA) positioned at the end of the circuit. The extracted air sample underwent analysis of water vapor pressure using an RH-100 device (Sable System, Las Vegas, NV, USA). The humid air was subsequently passed through a drying column filled with >99.5% CaSO_4_ (Drierite^®^) to remove humidity and enable an analysis of dry air concentration.

The concentrations of O_2_ and CO_2_ were analyzed from the dry air sample using paramagnetic (PA-10, Sable System, Las Vegas, NV, USA) and infrared (CA-10, Sable System, Las Vegas, NV, USA) analyzers, respectively. A universal interface (UI-3, Sable System, Las Vegas) was connected to the flowmeters and analyzers to record the signals at a frequency of one record per second. The signals from the analyzers and flowmeters were extracted using ExpData software v.1.9.22 (Sable System, Las Vegas, NV, USA).

The atmospheric air was conducted using a diaphragm pump, and O_2_ and CO_2_ concentrations were measured to establish the baseline concentrations.

### 2.2. General Calculations

The calculations employed in this study for simulating gas injection dynamics and data computation were based on the methods described by Ligton [15] and McLean and Tobin [16] for an open-circuit system operating under negative pressure.

The correction of the outgoing flow (Fout) via barometric (BP) and water vapor pressure (WVP) was calculated as $Fout={Fout}_{\left(dyl\right)}\;*\;\frac{BP}{BP-WVP}$, where Fout_(dyl)_ is the outgoing flow of wet air. The ingoing flow (Fin) was determined using the nitrogen correction factor via the equation $Fin=Fout\;*\left(\frac{N_{2}in}{N_{2}out}\right)$, where $N_{2}=100-O_{2}-{CO}_{2}$, disregarding the minor components of atmospheric air (e.g., Ag, CO, H_2_, CH_4_, etc.).

The volume of gases (L/min) was determined by multiplying the airflow and their respective gas concentration: ${VCO}_{2}out=Fout\times {CO}_{2}out$, ${VCO}_{2}in=Fin\times {CO}_{2}in$, and similarly for oxygen. The oxygen consumption (VO_2_) was calculated from the volumetric difference between ingoing and outgoing gases: ${VO}_{2}=Fin\times O_{2}in-Fout\times O_{2}out$. CO_2_ production (VCO_2_) was determined as ${VCO}_{2}=Fout\times {CO}_{2}out-Fin\times {CO}_{2}in$.

During the recovery test, the volume of injected CO_2_ (VCO_2_inj) was computed as ${VCO}_{2}inj=Finj\times {CO}_{2}inj$, where Finj is the controlled injection flow, and CO_2_inj is the known concentration of tested gas.

The HP was calculated based on the volumes of gas exchange using Brower’s fundamental equation [1]: $HP\left(kcal\right)=3.866\times {VO}_{2}+1.200\times {VCO}_{2}$.

### 2.3. Simulation of the Dynamic of Gas Injection in a Theoretical System

To perform the recovery test, a simulation was developed using Microsoft Excel spreadsheet (S1 File). The simulation was based on a theoretical system assuming constant flow with no significant resistances or leaks (Fin=Fout or $\frac{N_{2}in}{N_{2}out}=1$) and dry air passing through the system (WVP=0). This allowed us to describe the behavior of the injected CO_2_ concentration (CO_2_inj) over time (per minute). Parameters such as the volume of injected gas (Vinj), Finj, and CO_2_inj concentration were kept constant but could be modified for testing other gases based on this study’s objectives, system characteristics, and simulated scenarios. Additional calculations are detailed in Table 1.

**Figure 2:**
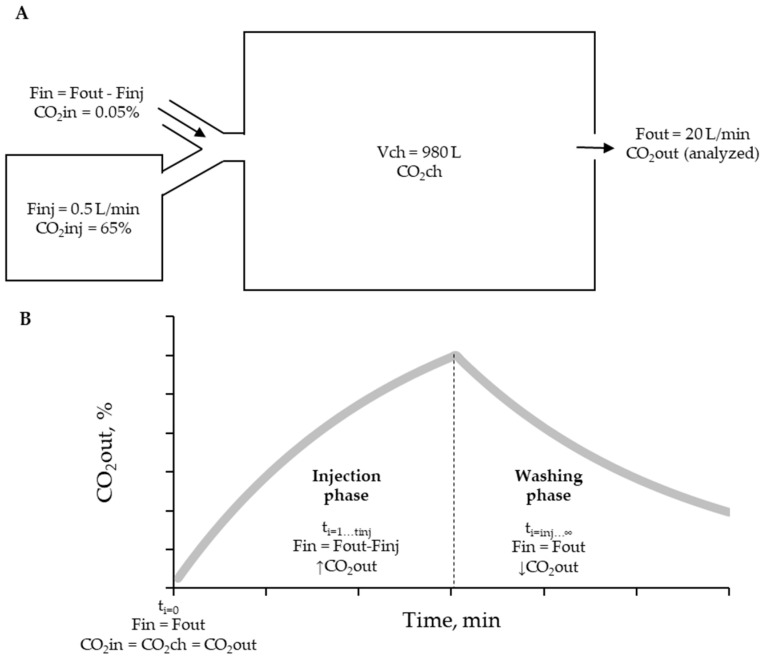
(**A**). Illustrative scheme of the recovery procedure with an injection of a known gas concentration (65% CO_2_). Fin: ingoing flow. Fout: outgoing flow. Finj: pure gas injection flow. V_CH_: chamber volume. Vbag: volume of the bag that contains tested gas. → airflow direction. (**B**). Phases of CO_2_ recovery test and CO_2_out behavior defined by the simulation. The tinj differentiates the injection and washing phases.

The simulation was conducted to determine the volume of CO_2_ in three compartments: (1) ingoing volume (VCO_2_in), (2) CO_2_ volume in the chamber (VCO_2_ch), and (3) outgoing volume (VCO_2_out) (Figure 2A). The simulation consisted of two phases: the injection phase (when Finj > 0 and ti < tinj) and the washing phase (when Vinj was empty at ti > tinj and Finj = 0) (Figure 2B).

At the start of the simulation (ti = 0), representing the absence of gas injection or baseline condition, the concentration of CO_2_ in all compartments was equal to the atmospheric air concentration. The volumetric content of each compartment was established as follows:

$${{VCO}_{2}}_{ti=0}=\left\{\begin{array}{c} V{CO}_{2}in\;\left(L/min\right)=Fout\times {CO}_{2}in\;;\;Finj=0;\;Fout=Fin\\ V{CO}_{2}ch\;\left(L\right)=Vch\times {CO}_{2}in\\ V{CO}_{2}out\;\left(L/min\right)=Fout\times {CO}_{2}out;\;{CO}_{2}in={CO}_{2}out \end{array}\right.$$

For the injection phases (ti = 1 to tinj), the following calculations were performed:

$${{VCO}_{2}}_{ti=1\ldots tinj}=\left\{\begin{array}{c} V{CO}_{2}in\;(L/min)=\left(Fout-Finj\right)\times {CO}_{2}in\\ V{CO}_{2}ch\;\left(L\right)={V{CO}_{2}ch}_{ti-1}+{VCO}_{2}{in}_{ti}+{VCO}_{2}{inj}_{ti}-{{VCO}_{2}out}_{ti-1};\;{VCO}_{2}{inj}_{ti}=Finj\times {CO}_{2}inj\;\\ V{CO}_{2}out\;\left(L/min\right)=Fout\times {{CO}_{2}ch}_{ti};\;{CO}_{2}ch=\frac{{VCO}_{2}{ch}_{ti}}{Vch} \end{array}\right.$$

In the above equations, VCO_2_ch at t = 1 was calculated by summing the volume of CO_2_ in the chamber (VCO_2_ch) at t = i − 1, the VCO_2_in at t = i, and the VCO_2_inj at t = i, and then subtracting the VCO_2_out at t = i − 1. The VCO_2_out at t = i was used to determine the fractional concentration of CO_2_ in the chamber (CO_2_ch) at t = i, calculated as ${CO}_{2}ch=\frac{{VCO}_{2}ch}{Vch}$.

During the washing phase (ti = tinj + 1 to infinity), the CO_2_ volumes were calculated as follows:

$${{VCO}_{2}}_{ti=tinj+1-\to \infty }=\left\{\begin{array}{c} V{CO}_{2}in\;(\frac{L}{min})=Fin\times {CO}_{2}in\\ V{CO}_{2}ch\;\left(L\right)={V{CO}_{2}ch}_{ti-1}+{VCO}_{2}{in}_{ti}-{{VCO}_{2}out}_{ti-1}\\ V{CO}_{2}out\;\left(\frac{L}{min}\right)=Fout\times {{CO}_{2}ch}_{ti};\;{CO}_{2}ch=\frac{{VCO}_{2}{ch}_{ti}}{Vch} \end{array}\right.$$

The outputs of interest from the simulation over time included CO_2_out, the differential volume of CO_2_ (${∆{VCO}_{2}}_{ti}={{VCO}_{2}out}_{ti}-{{VCO}_{2}in}_{ti}$), and the cumulative differential volume of CO_2_ ($Cumulative{∆VCO}_{2}=\sum_{ti=0}^{i}{∆{VCO}_{2}}_{ti}$). These parameters, calculated per minute, were used for comparing the result of each chamber.

### 2.4. Recovery Test Procedure

The recovery test was conducted in four respirometry chambers to assess the accuracy and precision of the system by injecting a known concentration of CO_2_ and measuring the rate of gas ingoing and outgoing over time. The recovery protocol was designed with the following considerations: (i) continuous monitoring and control of the gas concentration and injection flow rate at every time unit (each second), (ii) ensuring that the concentration and injection flow rate fall within the expected range of metabolic rates observed in animal trials, and (iii) comparing the simulated flow rate of CO_2_ with the observed rate in each chamber to evaluate the accuracy and precision of the system.

A non-diffusion medical bag (Jiangsu Yuyue Medical Equipment & Supply Co., Ltd., Nanjing, China) was used with a capacity to store 30 L of a known CO_2_ concentration (65% analytical CO_2_ and 35% compressed nitrogen, standard gas mixture with guaranteed concentration, Code: ONU-1013, White Martins, SP, Brazil). The bag was connected to a micro-diaphragm pump with a pressure of 90 kPa (CTS Parker Hannifin, Cleveland, OH, USA). It was used to inject the gas into the chamber at a controlled injection flow rate of Finj = 0.5 L/min, which was monitored using a rotameter (Figure 1 and Figure 2A). After that, the data collection was started by 60 min (injection time) up to the bag empty and continued for another 60 min without injection (washing time). This process was repeated three times in each chamber, and the average of the three observations was compared with the expected behavior of CO_2_out, ΔCO_2_out, and Cumulative ΔCO_2_ obtained from the simulation at each time point (*t*i).

### 2.5. Data Analyses and Recovery Index Calculation

The data analysis for the recovery test followed the same calculation procedures as described in the simulation. Data was recorded at one-second intervals and then averaged every minute. Several parameters were evaluated to assess the accuracy of the system in each chamber, including the fractional concentration of CO_2_out, ΔVCO_2_, and cumulative ΔVCO_2_. The error (ε) and residual standard deviation (RSD) were calculated for each chamber and minute to evaluate the results.

The error (ε) was calculated as the difference between the observed and expected (simulated) value using the following equation:

$$\varepsilon \left(k\right)=k_{observed}-k_{expected}$$

The residual standard deviation (RSD) was determined by taking the square root of the sum of squared differences between the observed and expected values, divided by the sample size (n), as follows:

$$RSD\left(k\right)=\sqrt{\sum \frac{{\left(k_{observed}-k_{expected}\right)}^{2}}{n}}$$

In each chamber, both ε and RSD were calculated for CO_2_out(*t*i), ΔVCO_2_, and Cumulative ΔVCO_2_.

Additionally, the recovery rate was determined by comparing the observed volume of CO_2_ (∆VCO_2_ observed) with the expected volume of CO_2_ (∆VCO_2_ expected) in each chamber. The recovery rate was calculated over a period of 120 min using the following formula:

$$Recovery\;rate=\frac{\sum_{i=0}^{120}∆{VCO}_{2}observed}{\sum_{i=0}^{120}∆{VCO}_{2}expected}$$

Here, k represents CO_2_out(*t*i), ΔVCO_2_, or Cumulative ΔVCO_2_. The observed values were recorded in each chamber, while the expected values were obtained through the simulation.

### 2.6. The Procedure of Data Computing of a Multi-Chamber IC System

For this procedure, we used the IC data report presented by Camargos et al. [17] on broiler chickens to illustrate the step-by-step calculations involved in data management. The calculation spreadsheet developed in MS Excel containing this data is available as Supplementary Material (S2 File).

**Figure 3:**
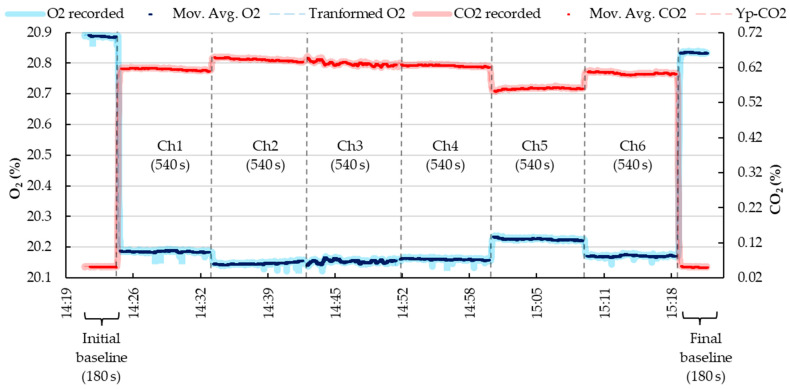
Example of recording, transforming, and filtering the gas concentration (O_2_—oxygen and CO_2_—dioxyde of carbon) sequence in a multi-chamber IC system. The black dashed line represents the recording time limit between chambers and the baseline.

The signals extraction of the fractional concentration of O_2_ and CO_2_, Fout (of each chamber), BPA, and WVP, exported one data per second to MS Excel. Since the experimental setup involved a multi-chamber IC system, certain inputs were necessary to define the recording sequence of the analyzers, which was controlled by a multiplexer, and to enable automated data processing. These inputs were incorporated into the MS Excel spreadsheet. Specifically, the programmed time sequence in ExpeData was provided within the MS Excel spreadsheet. This sequence dictated the recording of gas concentrations during both the baseline and chamber measurement periods. In the example provided in S2 File, the program was set to include an initial and final baseline (each with a reading duration of 180 s) and to record the gas concentrations in each chamber between the baselines (each with a reading duration of 540 s). Consequently, a complete cycle of gas concentration readings for all chambers lasted for 60 min (Figure 3), and this cycle was looped continuously for 24 h.

Detailed information regarding the chamber codification, sequence of inputs, and additional parameters (e.g., chamber volume and the body weight of animals inside the chambers) can be found in the S2 File.

### 2.7. Oxygen and CO_2_ Signal Calibration

The initial step in conducting IC measurements involved verifying the recorded signals and ensuring consistent gas concentration values. To achieve this, the analyzers were calibrated at the beginning and end of each metabolic measurement period. In the study conducted by Camargos et al. [17], calibration procedure was performed daily.

The calibration procedure employed two gases with known certified concentrations: Gas A, which consisted of pure nitrogen with approximately 99.99% N_2_, 0% O_2_, and 0% CO_2_ (White Martins, Guarulhos, SP, Brazil), and Gas B, a standard mixture comprising 21% O_2_ and 1% CO_2_ (White Martins, SP, Brazil).

For each gas of interest (O_2_ and CO_2_), a calibration curve was generated by extrapolating the concentrations over time (*t*i) using the following equation:

$${CO}_{2}\;extrapolated\;for\;ti\;(for\;gas\;A\;or\;B)={CO}_{2}t0+\left({CO}_{2}tn-{CO}_{2}t0\right)\times \frac{ti-t0}{tn-t0}$$

Here, CO_2_*t*i represents the fractional concentration of Gas A or Gas B at time *t*i. At the same time, CO_2_*t*0 and CO_2_*t*n denote the concentrations recorded by the analyzer at the initial and final time points for Gas A and B, respectively. The extrapolated CO_2_ concentration for each ti and gas (A and B) was then linearized as follows:

$$slope\left(ti\right)=\frac{{(CO}_{2}concentration\;for\;gas\;A-{CO}_{2}concentration\;for\;gas\;B)}{\left(CO_{2}extrapolated\;at\;ti\;for\;gas\;A-{CO}_{2}extrapolated\;at\;ti\;for\;gas\;B\right)}$$

$$intercept\left(ti\right)={CO}_{2}concentration\;for\;gas\;A-slope(ti)\times {CO}_{2}extrapolated\;at\;ti\;for\;gas\;A$$

Finally, the calibrated signals and expression of the gas concentrations were represented as follows:

$${CO}_{2}calibrated\left(ti\right)=intercept\left(ti\right)+slope\times {CO}_{2}register(ti)$$

The same procedure was applied to calibrate the O_2_ signals. In O_2_ and CO_2_ measurements, the fractional concentrations derived from the calibration process were used for subsequent calculations.

### 2.8. Calibrated Fractional Concentration and Filtering

The fractional concentration obtained through calibration alone is insufficient for calculating heat production (HP). Therefore, the reliability of the IC measurements relies on the detection of metabolic signals by the system or the appropriate mathematical techniques applied to highlight these metabolic events.

The Bartholomew transformation [18] is a commonly used procedure for real-time gas exchange measurements [19]. This transformation is based on the relationship between Vch and Fout, representing the system’s ability to detect the metabolic signal or provide a delay for corrective action in its absence.

The concentrations of O_2_ or CO_2_ can be corrected by incorporating the exponential saturation of the chamber, which is dependent on Fout. The calibrated concentrations of O_2_ and CO_2_ at a specific time (*t*i) can be corrected using the following equation:

$${CO}_{2}(ti)\;transformed=\frac{{CO}_{2}calibrated\left(ti\right)-{CO}_{2}calibrated\left(ti-1\right)}{1-e^{-\frac{Fout\left(ti\right)}{Vch}\times ti}}$$

After applying the Bartholomew transformation, the CO_2_ concentration is transformed to the time *t*i. Since the signals were recorded every second, a moving average (n = 10) was employed as a criterion to reduce noise and synchronize the O_2_ and CO_2_ analyzer signals.

$${CO}_{2}\left(t=0\ldots n\right)=\frac{1}{n}\sum_{i=0}^{n}{CO}_{2}(ti)transformed$$

The same procedure was conducted for O_2_. The transformed and filtered signals were then utilized to calculate HP based on the equations described by Lighton [15] and Gerrits et al. [9].

The detailed step-by-step procedure for a multi-chamber IC system can be shown in detail in the S2 File.

## 3. Results

### 3.1. Results of the Recovery Test

**Figure 4:**
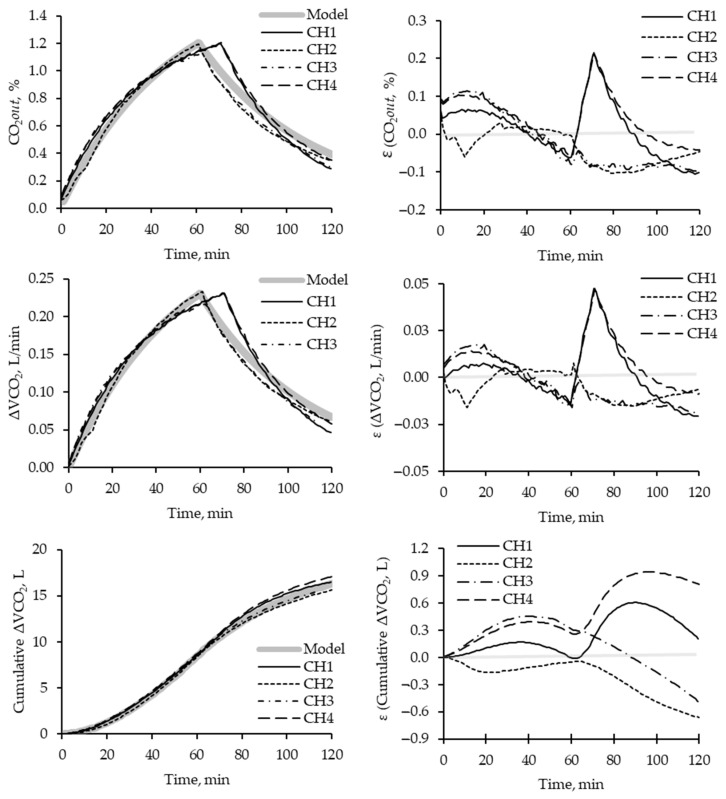
Dynamic (per minute) of the injection of known concentration of CO_2_ injected on each chamber and description of parameters of CO_2_out(*t*i), ΔVCO_2_, or Cumulative ΔVCO_2_, and the error calculated for each time. Each line represents the behavior of each chamber (CHn, where n refers to different chambers). The shadow line describes the expected results per unit of time according to the simulation.

Figure 4 illustrates the minute-by-minute dynamic behavior of each chamber. All chambers exhibited similar behavior for all parameters compared to the simulated model. However, comparing the behavior between chambers throughout the assay, chambers 2 and 3 demonstrated distinct patterns and exhibited deviations from chambers 1 and 4. Chamber 2 displayed greater consistency and closely adhered to the expected behavior curve more closely than the other chambers.

The results from the injection phase revealed that chambers 2 and 3 reached their *t*inj values at approximately 60 min, consistent with the expected time for completing gas injection. However, chambers 1 and 4 exhibited delays of around 15 min, taking longer than anticipated.

During the injection period, all chambers exhibited slightly higher values for CO_2_out concentration and ΔVCO_2_ than expected. Additionally, the washing period showed greater variation in CO_2_out(ti) parameters, ΔVCO_2_, and Cumulative ΔVCO_2_.

In general, a 1% variation in CO_2_ concentration resulted in a 0.05 L/min deviation in the volumetric ΔVCO_2_ above the expected value. However, this did not significantly impact the cumulative volumetric difference of CO_2_, and it is unlikely to pose a problem during animal experimentation, as it only resulted in less than 1 L of ΔVCO_2_ above the expected value.

Table 2 presents each chamber’s recovery rates and relative standard deviation (RSD). As mentioned earlier, chambers 2 and 3 exhibited similar behaviors with higher RSD (ΔVCO_2_) and lower RSD (Cumulative ΔVCO_2_) than chambers 1 and 4. Consequently, chambers 2 and 3 had recovery rates below 1, while chambers 1 and 4 had recovery rates above 1.

### 3.2. IC Data Computing and Filtering

The computation of signals began by calibrating the individual signals of O_2_ and CO_2_ in the function of time. The analysis of gas A, with a certified concentration of 100% N_2_, 0% O_2_, and 0% CO_2_, revealed the initial values of 0.008% O_2_ and 0.004% CO_2_ at the start of the measurement period and final values of 0.108% O_2_ and 0.007% CO_2_ at the end of the assay. Conversely, the analyzed concentration for gas B, with a certified concentration of 21% O_2_ and 1% CO_2_, showed the initial values of 21.14% O_2_ and 1.021% CO_2_ at the start of the measurements and the final values of 20.96% O_2_ and 0.982% CO_2_ at the end of the measurements (S2 File, see INPUTS sheet). These results yielded an average slope of 1.0001 and an average intercept of −0.058 for the O_2_ calibration curve in the function of time. As for the CO_2_ calibration curve in the function of time, an average slope of 1.004 and an average intercept of −0.006 were observed. The calibration curve was extrapolated per unit of time and is provided in the S2 File (see DATABASE sheet).

Once the calibrated signals for both gases were obtained, these were transformed and filtered to facilitate the calculation of gas exchange volumes and heat production. These procedures were performed for individual data and expressed per minute for each chamber (S2 File, see GAS EXCHANGE sheet).

**Figure 5:**
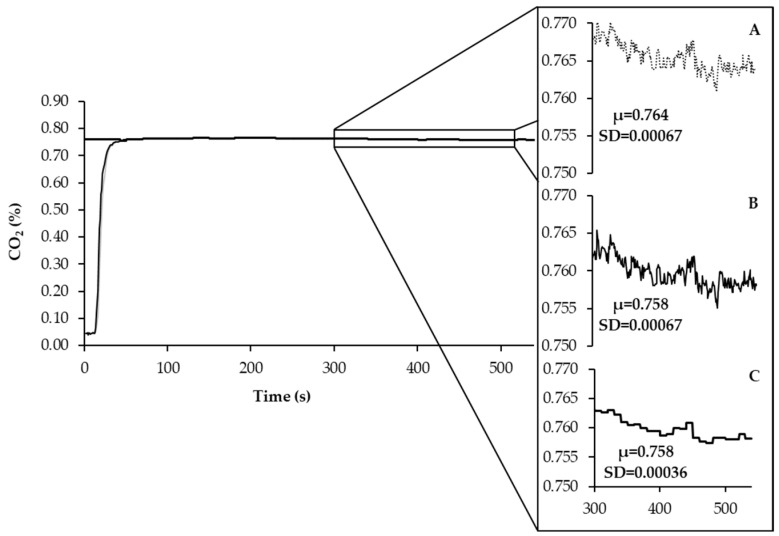
The curve of recorded signal (**A**) calibration and Bartholomew transformation applied to individual data (**B**) and filtered data with moving average (**C**) of the fractional concentration of CO_2_. In the same way, it was applied to O_2_.

As shown in Figure 5, the average fractional concentration of CO_2_, after calibration and Bartholomew transformation, exhibited a slight reduction. This reduction can be attributed to the volumetric contribution of CO_2_ within the chamber, which depends on Vch and the CO_2_out measurements recorded by the analyzer. At this stage, the variation observed in the recorded and calibrated-transformed data was comparable. To further mitigate noise in the data while preserving the average of the previously calibrated-transformed data, a moving average (n = 10) was applied.

## 4. Discussions

The recovery test results indicate that all chambers exhibited similar behaviors compared to the simulated model in terms of dynamic parameters. However, specific differences were observed among the chambers throughout the assay, particularly in the CO_2_ concentration, volume injection, washing, and cumulative volume of CO_2_ passing through each chamber. These differences in behavior can be attributed to variations in chamber geometry, as well as factors influencing gas exchange, such as the efficiency of gas mixing inside the chamber and the performance of the heater–cooler system [15,16]. The presence of a temperature-controlled system inside the chamber is especially important and should be weighted and taken into consideration during calibration trials preceding each metabolic assay.

The observed variations in the delay to reach the target *t*inj among the chambers can be attributed to differences in the accuracy of filling the non-diffusion bag used in the assays [12]. As the Vinj was similar across chambers and repetitions, accurately measuring the exact volume becomes challenging and can impact the expansion of the tested gas.

During the injection period, an interesting finding was that all chambers exhibited slightly higher CO_2_out concentrations and ΔVCO_2_ values than expected. These variations in ΔVCO_2_ are attributed to airflow (Finj or Fout), which can be calibrated using a flowmeter to provide greater precision during animal experimentation [15]. These observations suggest the presence of some inefficiencies or measurement errors in capturing and recording gas exchange data. However, the impact of these variations on the overall cumulative volumetric difference of CO_2_ was minimal, resulting in less than 1 L of ΔVCO_2_ above the expected value. Therefore, these discrepancies are unlikely to significantly affect the results’ accuracy or pose problems during animal experimentation.

Each chamber’s recovery rates and RSDs provide insights into the system’s performance. As McLean and Tobin [16] recommended, the recovery rates fall within the system inefficiency range [16]. An acceptable recovery rate range of 3% to 8% (0.92 to 1.08) is considered normal.

The slight reduction observed in the average fractional concentration of CO_2_ after calibration and Bartholomew transformation can be attributed to the volumetric contribution of CO_2_ within the chamber, which depends on Vch, in addition to the CO_2_out measurements recorded by the analyzer. The variation observed in the recorded and calibrated-transformed data was similar at this stage. To further enhance data quality by reducing noise while preserving the average of the previously calibrated–transformed data, a moving average (n = 10) was applied.

The computation of IC data follows a comprehensive methodology described by Lighton [15] and Gerrits et al. [9]. This approach is applicable when working with analyzers and chambers that have undergone thorough checking and calibration. It is crucial to ensure that the chambers are defect free or have passed a recovery test to assess their suitability. Additionally, the output signals of the analyzer should accurately represent the temporal variation observed during animal experimentation. Therefore, the periodic calibration of the analyzer, recorded signal verification, and application filtering techniques are recommended to obtain coherent gas concentration values while minimizing noise. The calibration curves obtained allow for the accurate transformation and subsequent filtering of the signals to calculate gas exchange volumes and heat production.

The findings from this study align with previous literature. Lighton [15] and Gerrits et al. [9] have emphasized the importance of accurate calibration and periodic verification of signals in computing IC data. Also, this present study contributes to the existing body of literature by demonstrating the importance of recovery tests in assessing the performance of chambers and the accuracy of gas exchange measurements. The variations observed among chambers highlight the need to carefully consider chamber geometry and other factors influencing gas exchange when designing experiments and interpreting results.

It is worth noting that this study has certain limitations. Using a specific animal model and experimental setup may restrict the generalizability of the results to other systems or species. Additionally, the analysis focused on CO_2_ measurements, and further investigations may be necessary to evaluate the behavior of other gases or parameters and on the other IC system kinds.

## 5. Conclusions

In conclusion, this study provides valuable insights into the behavior and performance of chambers used for measuring gas exchange in animal experimentation. The observed variations among chambers and deviations from the simulated model highlight the importance of considering chamber-specific factors and calibration procedures in order to obtain accurate and reliable data.

The findings emphasize the significance of calibration and filtering techniques in improving the accuracy of indirect calorimetry (IC) measurements. This study lays the groundwork for enhancing gas exchange measurements’ overall precision and reliability in future research by addressing the challenges associated with chamber behavior and data processing.

The adaptable nature of this methodology allows for its application in different IC systems and research objectives, making it a valuable tool for researchers in various fields. Moreover, the MS Excel spreadsheet provided in this study can be customized to meet specific research needs or serve as a practical teaching resource.

## Figures and Tables

**Table 1:** Parameters and calculations used for the simulation of recovery test over time (ti).

Variable		Description	Parameter or Calculation	Units
Initial parameters for the simulation				
	Atmospheric CO_2_ or fractional ingoing concentration	CO_2_in	0.05	%
	Atmospheric oxygen or fractional ingoing concentration	O_2_in	21	%
	Outgoing airflow (dry air)	Fout_(ti)_	20	L/min
	Volume of injection	Vinj	30	L
	Fractional concentration of CO_2_ injected	CO_2_inj	65	%
	Injection flow for ti < tinj	Finj_(ti)_	0.5	L/min
Intermediate calculations for *t* = i				
	Injection time	tinj	Vinj/Finj = 60	min
	Ingoing volume of CO_2_	VCO_2_in_(ti)_	Fin_(ti)_×CO_2_in_ti_	L/min
	Injected volume of CO_2_	VCO_2_inj_(ti)_	Finj_(ti)_×CO_2_inj_ti_	L/min
	Volume of CO_2_ in the chamber	VCO_2_ch_(ti)_	VCO_2_ch_ti−1_ + VCO_2_in_ti_ + VCO_2_inj_ti_ − VCO_2_out_ti−1_	L
	Fractional concentration of CO_2_ in the chamber	CO_2_ch_(ti)_	VCO_2_ch/Vch	%
	Outgoing volume of CO_2_	VCO_2_out_(ti)_	Fout_ti_×CO_2_ch_ti_	L/min
Outputs				
	Fractional concentration of outgoing CO_2_	CO_2_out_(ti)_	CO_2_ch_ti−1_	%
	Differential volume of CO_2_	ΔVCO_2_	VCO_2_out_ti_ − VCO_2_in_ti_	L/min
	Cumulative volume of differential CO_2_	Cumulative ΔVCO_2_	ΣΔVCO_2_ti→∞	L

**Table 2:** Volumetric recovery of CO_2_ (VCO_2_ recovered (*t*i→120), L), recovery rate, and residual standard deviation calculated for the fractional concentration of CO_2_, the volumetric difference of CO_2_ (ΔVCO_2_) and cumulative volumetric difference of CO_2_ (Cumulative ΔVCO_2_) describe for each chamber (CHn) during the recovery test.

Chamber	VCO_2_ Recovered (*t*i→120), L	Recovery Rate	RSD_(%CO2out)_	RSD_(ΔVCO2)_	RSD_(Cumulative ΔVCO2)_
CH_1_	16.51	1.021	0.419	0.084	0.016
CH_2_	14.87	0.920	0.730	0.172	0.032
CH_3_	15.09	0.933	0.476	0.115	0.020
CH_4_	17.09	1.057	0.693	0.081	0.015

## Data Availability

The data used on this article is available on the supplementary material.

## Supplementary Materials

The following supporting information can be accessed at: https://www.mdpi.com/article/10.3390/ani13162675/s1, S1: Spreadsheet of the dynamic of simulation for CO_2_; S2: Spreadsheet for data computing.
